# Adverse childhood experiences, child behavioral health needs, and family characteristics associated with the presence of a firearm in the home: a survey of parents in Chicago

**DOI:** 10.1186/s40621-023-00444-7

**Published:** 2023-07-24

**Authors:** Megan M. Attridge, Marie E. Heffernan, Anne Bendelow, Carly G. Menker, Matthew M. Davis, Karen Sheehan

**Affiliations:** 1 Division of Pediatric Emergency Medicine, Department of Pediatrics, Northwestern Feinberg School of Medicine, Ann & Robert H. Lurie Children’s Hospital of Chicago, 225 East Chicago Avenue, Box 62, Chicago, IL 60611 USA; 2 Department of Pediatrics, Northwestern Feinberg School of Medicine, Smith Child Health Outcomes, Research, and Evaluation Center and Stanley Manne Children’s Research Institute, Ann & Robert H. Lurie Children’s Hospital of Chicago, 225 East Chicago Avenue, Box 157, Chicago, IL 60611 USA; 3 Department of Data Analytics and Reporting, Ann & Robert H. Lurie Children’s Hospital of Chicago, 680 North Lake Shore Drive 13-002D;307, Chicago, IL 60611 USA; 4 Ann & Robert H. Lurie Children’s Hospital of Chicago, 225 East Chicago Avenue, Box 157, Chicago, IL 60611 USA; 5 Division of Advanced General Pediatrics and Primary Care, Department of Pediatrics, Northwestern Feinberg School of Medicine, Ann & Robert H. Lurie Children’s Hospital of Chicago, 225 East Chicago Avenue, Box 1, Chicago, IL 60611 USA; 6 Division of Emergency Medicine, Department of Pediatrics, Northwestern Feinberg School of Medicine, Ann & Robert H. Lurie Children’s Hospital of Chicago, 225 East Chicago Avenue, Box 62, Chicago, IL 60611 USA

**Keywords:** Firearms, Violence, Accidental injuries, Suicide, Parents, Chicago

## Abstract

**Background:**

Firearm violence is the leading cause of pediatric mortality in the USA. The presence of a firearm in the home poses an immense risk to children with increased rates of suicide and unintentional injury by firearm. Recent literature has not explored child ACEs and child behavioral health needs with the presence of a firearm in the home. The objective of this study was to explore an association between these factors, parent health, family experience with firearm violence, and demographics, and the presence of a firearm in the home.

**Results:**

Overall, 382 of 1,436 (weighted to 22.0%) responding parents reported the presence of a firearm in the home. In an adjusted model, the odds ratio of firearm presence increased incrementally with a child’s increasing exposure to ACEs. Compared to a child in the household exposed to no ACEs, a child in the household exposed to two or more ACEs was associated with a 5.16 times higher odds of firearm presence in the home (95% confidence interval (CI) 2.92–9.10). Similarly, a child in the household who had used behavioral health services was associated with a 2.10 times higher odds of firearm presence in the home (95% CI 1.35–3.26), compared to a child in the household who had not. Presence of firearm in the home was also associated with higher household income, younger parent age (under 35 years), and male parent gender.

**Conclusions:**

Chicago parents have higher odds of reporting the presence of a firearm in the home when living in a household with a child exposed to ACEs and with behavioral health needs. These findings could inform future public health interventions and targeted safe storage messaging to prevent pediatric firearm injury in the home.

## Introduction

Pediatric firearm injury rates have steadily increased over the last decade, and in 2020, firearm injury became the leading cause of death of children and youth under 18 years of age (Centers for Disease Control and Prevention, National Center for Health Statistics 2021; Centers for Disease Control and Prevention: National Center for Injury Prevention and Control 2005). Youth firearm violence is unique to the USA; compared to youth in other high-income countries, US children experience rates of firearm suicide that are 8 times higher, and rates of unintentional injury death that are 10 times higher (Richardson and Hemenway 2011; Gardner et al. 2012). The financial burden of youth firearm violence is also significant, with an estimated $109 million spent annually on initial hospitalization costs for pediatric firearm injuries (Taylor et al. 2021).

The presence of a firearm poses significant risk to the children living in the home. Children as young as three years of age can pull the trigger of a gun (Naureckas et al. 1995). The rate of youth suicide is four times higher among children who live in a home with a firearm (Miller et al. 2007; Schaechter 2022). Similarly, the risk of death by unintentional firearm injury is four times higher for children who live in homes with firearms (Miller et al. 2001; Grossman et al. 2005; Monuteaux et al. 2019). The American Academy of Pediatrics advises that “the safest home for a child or adolescent is one without firearms”; however despite this recommendation, an estimated one third of children in the USA live in a home with a firearm (Gardner et al. 2012; Schaechter 2022).

Adverse childhood experiences (ACEs) are negative or traumatic experiences during childhood, such as witnessing violence, experiencing the divorce of a parent, or living in a household with someone who has a problem with alcohol or drugs (Felitti et al. 1998). Some measures of ACEs, such as the one used in the present study, also include being treated unfairly because of one’s race or ethnicity (Child and Adolescent Health Measurement Initiative (CAHMI) 2018; Health Resources and Services Administration’s Maternal Child Health Bureau 2020). ACEs have been shown to have detrimental effects on health from childhood to adulthood (Felitti et al. 1998). Children who experience ACEs are also at increased risk of violence victimization and perpetration (Burke et al. 2022). While prior literature suggests that firearm ownership is higher among non-Hispanic White individuals, males, individuals with higher annual income, individuals with lower education, and adults aged 65 and up (Oraka et al. 2019; Parker et al. 2017), few studies have explored child, parent, and family characteristics, such as exposure to ACEs, as predictors of the presence of a firearm in the home. The primary objective of this study was to explore an association between a child’s exposure to ACEs and the presence of a firearm in the home. Prior work has identified potential associations with firearm violence and poor physical health (Hempstead et al. 2013) and exposure to firearm violence (Goldstick et al. 2017); furthermore, youth firearm access has been associated with increased violence risk and behavioral health issues (Sigel et al. 2019). Therefore, we also sought to explore associations between a child’s use of behavioral health services, parent health, and history of firearm violence personally affecting the family, as well as parent and family demographics with the presence of a firearm in the home.

## Results

### Patient characteristics

Of the 2,181 eligible probability-based participants who received the survey, 1,231 responded for a response rate of 56.4%. Additional respondents from non-probability-based panels also completed the survey, for a total of 1,505 responses. After participants with missing data for our selected variables were excluded, the sample size was *N* = 1,436. Responding parents were female (weighted 56.4%, 95% CI 52.5–60.4), over 35 years of age (weighted 67.0%, 95% CI 63.5–70.4), Hispanic (weighted 39.3%, 95% CI 35.4–43.1) ethnicity, received college graduate education or above (weighted 39.2%, 95% CI 35.8–42.7), had a household income between 100 and 399% of the federal poverty line (weighted 41.7, 95% CI 37.9–45.5) (Table 1).

**Table 1:** Characteristics of parents reporting firearms in the home vs no firearms in the home

	Total Weighted % (95% CI)	Firearms in the Home Weighted % (95% CI)	No firearms in the home Weighted % (95% CI)
All	100	22.0 (19.1–24.9)	78.0 (75.1–80.9)
Child ACEs			
2+ ACEs	12.1 (9.8–14.3)	53.8 (44.1–63.5)	46.2 (36.5–55.9)
1 ACE	25.9 (22.7–29.2)	23.2 (17.2–29.2)	76.8 (70.8–82.8)
No ACEs	62.0 (58.4–65.6)	15.3 (12.2–18.4)	84.7 (81.6–87.8)
Has your family been personally affected by firearm violence?			
Yes	21.5 (18.4–24.6)	34.2 (26.9–41.5)	65.8 (58.5–73.1)
No	78.5 (75.5–81.6)	18.7 (15.5–21.8)	81.3 (78.2–84.5)
Parent health			
Better health	65.9 (62.2–69.6)	27.0 (23.1–30.8)	73.0 (69.2–76.9)
Worse health	34.1 (30.4–37.8)	12.4 (8.2–16.6)	87.6 (83.4–91.8)
Has your child ever used specialty behavioral health services?			
Yes	36.7 (33.1–40.4)	35.6 (30.1–41.2)	64.4 (58.8–69.9)
No	63.3 (59.6–66.9)	14.1 (10.9–17.2)	85.9 (82.3–89.1)
Parent gender			
Male	43.6 (39.6–47.5)	35.3 (29.3–41.3)	64.7 (58.7–70.7)
Female	56.4 (52.5–60.4)	11.7 (9.3–14.1)	88.3 (85.9–90.7)
Parent current age			
Over 35 years	67.0 (63.5–70.4)	19.7 (16.3–23.1)	80.3 (76.9–83.7)
35 years and under	33.0 (29.6–36.4)	26.7 (21.3–32.1)	73.3 (67.9–78.7)
Parent race & ethnicity			
Asian/other race, non-Hispanic	9.4 (7.3–11.5)	15.9 (7.3–24.5)	84.1 (75.5–92.7)
Black, non-Hispanic	19.9 (16.9–22.9)	19.8 (12.8–26.7)	80.2 (73.3–87.2)
Hispanic	39.3 (35.4–43.1)	16.3 (11.8–20.9)	83.7 (79.1–88.2)
White, non-Hispanic	31.4 (28.3–34.6)	32.3 (27.2––37.4)	67.7 (62.6–72.8)
Parent education			
College graduate or above	39.2 (35.8–42.7)	28.7 (24.7–32.7)	71.3 (67.4–75.3)
Some college or technical school	23.3 (20.3–26.3)	18.7 (12.9–24.4)	81.3 (75.6–87.1)
High school or below	37.4 (33.4–41.5)	17.1 (11.6–22.6)	82.9 (77.4–88.4)
Household Income as % of FPL			
400% or greater FPL	35.7 (32.3–39.1)	33.4 (28.3–38.6)	66.6 (61.4–71.7)
100–399% FPL	41.7 (37.9–45.5)	19.1 (14.8–23.4)	80.9 (76.6–85.2)
Less than 100% FPL	22.6 (19.3–25.9)	9.2 (3.8–14.7)	90.8 (85.3–96.2)

*ACEs* Adverse Childhood Experiences, *FPL* Federal Poverty Level

Overall, 382 of 1,436 responding parents reported a firearm in the home (weighted 22.0%, 95% CI 19.1–24.9). Nearly a quarter (weighted 21.5%, 95% CI 18.4–24.6) of parents reported their family had personally been affected by firearm violence. Most responding parents reported “better health” (weighted 65.9%, 95% CI 62.2–69.6). Over a third (weighted 36.7%, 95% CI 33.1–40.4) reported a randomly selected child had ever used a behavioral health service. Parents reported the ACE exposure of a randomly selected child in the household: 25.9% (weighted, 95% CI 22.7–29.2) experienced one ACE, and 12.1% (weighted, 95% CI 9.8–14.3) experienced two or more ACEs (Table 1). The most frequently experienced ACE was “parent or guardian divorced or separated,” (weighted 15.5%, 95% CI 13.0–18.0) followed by “treated or judged unfairly because of his or her race or ethnic group” (weighted 12.1%, 95% CI 9.6–14.6) (Table 2).

**Table 2:** Adverse childhood experience frequencies

Adverse childhood experience	Weighted % of children exposed to ACE (95% CI)
Parent or guardian divorced or separated	15.5 (13.0–18.0)
Treated or judged unfairly because of his or her race or ethnic group	12.1 (9.6–14.6)
Was a victim of violence or witnessed violence in the neighborhood	6.8 (5.0–8.7)
Lived with anyone who was mentally ill, suicidal, or severely depressed	6.3 (4.7–8.0)
Lived with anyone who had a problem with alcohol	6.0 (4.4–7.5)
Parent or guardian served time in jail	6.0 (4.3–7.7)
Saw or heard parents or adults slap, hit, kick punch one another in the home	5.7 (4.1–7.2)
Parent or guardian died	4.9 (3.4–6.4)

Weighted frequency of Adverse Childhood Experiences (ACEs) experienced by a randomly selected child in the household

*CI* Confidence Interval

*ACE* Adverse Childhood Experience

### Factors associated with the presence of a firearm in the home

In the unadjusted model, the presence of a firearm in the home was significantly associated with child exposure to ACEs (with one ACE OR 1.67, 95% CI 1.10–2.53 and two or more ACEs OR 6.44, 95% CI 4.07–10.19 compared to no ACEs); a parent reporting their family had personally been affected by firearm violence (OR 2.27, 95% CI 1.55–3.33); a parent reporting “better health” (OR 2.61, 95% CI 1.70–4.01 compared to worse health); and a child using specialty behavioral health services (OR 3.39, 95% CI 2.37–4.84). When examining sociodemographic characteristics of parent and households, in the unadjusted model, the presence of a firearm in the home was significantly associated with male parent gender (OR 4.11, 95% CI 2.89–5.87), higher parent education (college graduate or above OR 1.95, 95% CI 1.26–3.01, compared to high school education or below), and incrementally with household income (with household income of 100–399% FPL OR 2.32, 95% CI 1.14–4.70, and household income of 400% or greater FPL OR 4.93, 95% OR 2.48–9.82 compared to less than 100% FPL). Parent age over 35 years, and parent race and ethnicities of Hispanic, non-Hispanic Black, Asian and other race compared to non-Hispanic White race and ethnicity were associated with lower odds of the presence of a firearm in the home in the unadjusted analysis (Table 3).

**Table 3:** Regression analysis of family characteristics associated with firearms in the home

	Firearm in the Home Odds ratio (95% CI)	Firearm in the Home Adjusted ODDS ratio (95% CI)
Child ACEs		
2+ ACEs	*6.44 (4.07–10.19)	*5.16 (2.92–9.10)
1 ACE	*1.67 (1.10–2.53)	*1.87 (1.14–3.05)
No ACEs	Reference	Reference
Child age		0.98 (0.93–1.02)
Has your family been personally affected by firearm violence?		
Yes	*2.27 (1.55–3.33)	1.55 (0.98–2.45)
No	Reference	Reference
Parent health		
Better health	*2.61 (1.70–4.01)	1.64 (0.98–2.73)
Worse health	Reference	Reference
Has your child ever used specialty behavioral health services		
Yes	*3.39 (2.37–4.84)	*2.10 (1.35–3.26)
No	Reference	Reference
Parent gender		
Male	*4.11 (2.89–5.87)	*3.04 (2.02–4.57)
Female	Reference	Reference
Parent current age		
Over 35 years	*0.67 (0.47–0.96)	*0.59 (0.38–0.91)
35 years and under	Reference	Reference
Parent race & ethnicity		
Asian/other race, non-Hispanic	*0.40 (0.20–0.79)	0.68 (0.31–1.52)
Black, non-hispanic	*0.52 (0.31–0.85)	1.08 (0.55–2.13)
Hispanic	*0.41 (0.27–0.61)	0.71 (0.40–1.28)
White, non-Hispanic	Reference	Reference
Parent education		
College graduate or above	*1.95 (1.26–3.01)	0.79 (0.41–1.55)
Some college or technical school	*1.11 (0.65–1.91)	0.87 (0.45–1.70)
High school or below	Reference	Reference
Household income as % of FPL		
400% or greater FPL	*4.93 (2.48–9.82)	*4.26 (1.87–9.67)
100–399% FPL	*2.32 (1.14–4.70)	*2.46 (1.20–5.04)
Less than 100% FPL	Reference	Reference

Multivariable analysis exploring the association of family characteristics with the presence of a firearm in the home. This regression adjusts for child ACEs for a randomly selected child in the household, the age of the randomly selected child, family affected by firearm violence, parent health, child behavioral health needs, parent gender, parent current age, parent race & ethnicity, parent education, household income

*CI*: Confidence Interval

*ACEs*: Adverse Childhood Experiences

*FPL*: Federal Poverty Level

*Indicates significance (95% confidence interval does not cross 1)

In an adjusted model that included all predictors and demographic variables, parents living with a child who experienced one ACE had 1.87 higher adjusted odds of reporting the presence of a firearm in the home (95% CI, 1.14–3.05) compared to no ACEs; living with a child who experienced two or more ACEs had 5.16 higher adjusted odds of reporting a firearm in the home compared to no ACEs (95% CI, 2.92–9.10). Reporting a child having ever used specialty behavioral health services was associated with 2.10 times higher adjusted odds of reporting the presence of a firearm in the home (95% CI, 1.35–3.26). A parent reporting their family had personally been affected by firearm violence, and better parent health was no longer significant in the adjusted model (Table 3). Male parent gender (aOR 3.04, 95% CI 2.02–4.57) compared to female parent gender, and higher income (aOR 2.46, 95% CI 1.20–5.04 for household income 100–399% FPL; and aOR 4.26, 95% CI 1.87–9.67 for household income 400% and greater FPL compared to less than 100% FPL) remained associated with the presence of a firearm in the home. Parent age over 35 years also remained associated with lower odds of firearm presence in the home. Parent race and ethnicity and parent education were no longer associated with the presence of a firearm in the home in the adjusted model (Table 3).

In a post-hoc chi-square analysis among only respondents whose marital status was married, married males were significantly more likely to report having a firearm in the home compared with married females (33.6%, 95% CI 27.4–39.9 vs. 12.6%, 95% CI 9.8–15.3, *p* < 0.0001).

## Discussion

These survey results demonstrated an association between the presence of a firearm in the home with a child’s exposure to ACEs and a child’s use of behavioral health services. When evaluating sociodemographic characteristics, the presence of a firearm in the home was also associated with male gender, parent age 35 years and under, and increasing income.

This study supports similar work on family demographics and firearm ownership. About 22% of Chicago parents in this study reported the presence of a firearm in the home. This is less than the one third of homes in the USA estimated to have a firearm present in the home (Azrael et al. 2018). This may be explained by significant regional variation across the nation, with the highest firearm ownership reported in southern and rural regions of the country, compared to this single urban city study (Oraka et al. 2019; Parker et al. 2017; Connor 2005). Unadjusted analyses suggested higher presence of firearm in the home among non-Hispanic White race and ethnicity, similar to previous studies; (Miller et al. 2007; Parker et al. 2017) however, this association dissolved in the adjusted model. The lack of association with race and ethnicity may be unique to Chicago’s parent population or suggest that this model includes new variables associated with race and ethnicity that better predict the outcome of firearm presence in the home. While previous studies suggest that older adults are more likely to report gun ownership (Oraka et al. 2019; Parker et al. 2017), our study, which focused on parents, naturally excluded a significant portion of adults aged 65 years and older; this study demonstrated higher odds of reporting a firearm in the home among younger parents ages 35 years and under. Males were more likely to report the presence of firearm in their homes in this study which is consistent with multiple previous analyses (Oraka et al. 2019; Parker et al. 2017; Connor 2005). We also found that males were more likely to report firearms in the home than females regardless of marital status, suggesting that this discrepancy is not entirely driven by single male parents reporting firearm ownership, and among married households (92% of our married sample reported being heterosexual), males were more likely to report firearm ownership than females. These findings are like those of Coyne-Beasley et al. in their 2005 study of cohabitating partners which demonstrated a gender gap in the reporting of firearm ownership (Coyne-Beasley et al. 2005). Similar to prior work, our study also demonstrated an association with increasing income and the presence of firearm in the home (Oraka et al. 2019). Previous authors have postulated that this may be due, in part, to greater expendable income (Oraka et al. 2019; Kahan and Bramant 2003).

To our knowledge, this is the first study to demonstrate an association of child exposure to ACEs with the presence of a firearm in the home. ACEs are traumatic early events in a child’s life that can not only have ill-effects on their future health, but also place a child at increased risk of victimization and perpetration of violence in the future (CDC – Centers for Disease Control and Prevention 2022). Considering children who live in homes with firearms are at increased of unintentional injury, suicide, and homicide, children living in homes with firearms who have also experienced ACEs may experience even higher risk of harm from firearms. Furthermore, as screening for child ACEs has become more commonplace in some medical settings, identifying children who have experienced ACEs could provide a targeted opportunity to counsel on the safe storage of firearms. This study also found that a child’s utilization of behavioral health services was associated with firearm presence in the home. This is particularly important as access to firearms is a significant risk factor for youth suicide (Dahlberg et al. 2004). In a concerning study, Schnitzer et al. found that among youth who died by suicide, children who had previously talked about or threatened or attempted suicide were less likely to live in homes where firearms were stored in a locked location compared to those who had not talked about, threatened, or attempted suicide (Schnitzer et al. 2019). Our study further supports lethal means and safe firearm storage counseling to families, in particular those with children with behavioral health needs.

### Limitations

This study has several limitations. Primarily, the presence of firearm in a family’s home was self-reported. Considering Illinois state child access prevention laws, participants may have underreported firearm presence in the home for fear of repercussion. The nature of this observational study does not allow for assumptions of causation, but instead may be used to identify families at greater need of safe firearm storage counseling. Similarly, there is risk of ecological fallacy and results of this observational study should be interpreted with caution at the level of the individual family. For our model, we chose to include parental and family characteristics that may influence firearm presence in the home as parents are likely responsible for keeping firearms with the exception of child ACEs and child’s use of behavioral health services as these variables may reflect increased risk to the child living in a home with firearms. However, future work could explore how child demographics or parental ACEs associate with firearm presence in the home. Our survey tool, which allowed for brevity, presented challenges. For example, survey responses did not allow for in-depth responses to questions regarding the child’s use of behavioral health services, parent health, and history of firearm violence personally affecting the family. Furthermore, parent responses may not accurately reflect ACEs experienced or behavioral health services accessed by their children and adolescents without their knowledge. Additional research is needed to explore these variables in more detail. While non-response was rare in these survey results, this is an opportunity for bias to skew results. While not necessarily a limitation, these results were collected during the COVID-19 pandemic during which firearm sales rose rapidly (Schleimer et al. 2021), and more children experienced behavioral health concerns (Theberath et al. 2022). This context may have changed the family characteristics associated with firearm ownership compared with prior work. Furthermore, the association between a child using behavioral health services and the presence of the firearm in the home potentially becomes more important in the setting of rising firearm ownership and pediatric behavioral health needs, which could have important policy implications. Lastly, this study largely focuses on child adversity and risk; while it is important to understand risk factors for firearm exposure and injury, future work should examine child, parent, and family strengths as opportunities to protect children from firearm injury.

## Conclusion

Chicago parents have higher odds of reporting the presence of a firearm in the home when living in a household with a child exposed to ACEs or a child that has used behavioral health services. These findings are particularly important in the context of injury prevention, considering children experiencing ACEs or using behavioral health services may be at increased risk of experiencing firearm violence. These findings could inform future public health interventions and targeted safe storage messaging to prevent pediatric firearm injury in the home.

## Methods

### Study design and population

This survey study was conducted among adult Chicago parents between November 2020 and February 2021 through the Voices of Child Health in Chicago Parent Panel Survey (Wave 2). Parents were from all 77 neighborhoods in Chicago. Parents indicated their preferred method for completing surveys (online or phone) during their initial survey with the panel, and the majority (94%) of parents completed the survey online. Parent participants were first recruited from the probability-based VOCHIC panel and NORC’s AmeriSpeak panel. To ensure sufficient sample size to permit subgroup analyses, the probability sample was augmented by calibration-weighted, non-probability-based responses obtained through other vendor panels. A response rate (i.e., denominator) for the non-probability sample was unable to be measured because these panels administer opt-in online surveys.

To determine eligibility, parents completed a screener; parents were eligible if they were adults, 18 years of age and older, with at least one child under the age of 18 living in their household, and if they resided in Chicago. Eligible parents were invited to complete the survey which was administered by NORC at the University of Chicago, in English and Spanish, online and over the telephone based on parent preference. Participants were compensated between $5–15 to complete the survey. Participation was optional and voluntary. The Institutional Review Boards at Ann & Robert H. Lurie Children’s Hospital of Chicago and NORC determined that this study was exempt from human subject review. Participants who did not respond received email reminders.

### Parent survey

Wave 2 of the Voices of Child Health in Chicago Parent Panel Survey covered nine different themes, including child water safety, discrimination, child and parent physical and mental health (including child exposure to ACEs), COVID-19, vaccines, and firearms. The survey included 58 items in total (not including demographic items), with eight items pertaining to firearm safety and parent concerns about firearm violence. Parents also responded to items regarding basic demographic and family characteristics.

### Study measures

Our primary outcome measure was the presence of a firearm in the parent’s household (“Are there any guns in your home?” [response options: yes, no]) as a dichotomous variable. Our primary independent variable of interest was the exposure to ACEs of a randomly selected child in the home. The ACEs measure included the question prompt, “To the best of your knowledge, has your child ever experienced any of the following…” followed by a list of eight items (e.g., parent or guardian divorced or separated; parent or guardian died, see Table 2 for full items) and was from the 2018 National Survey of Children’s Health.(Child and Adolescent Health Measurement Initiative (CAHMI) 2018; Health Resources and Services Administration’s Maternal Child Health Bureau 2020) We transformed responses into a categorical variable (child experienced no ACEs, one ACE, two or more ACEs), adjusting for child age (as older children have more time to accumulate additional ACEs) in the multivariable analysis. We explored several additional independent variables of interest including the child’s use of behavioral health services (“When was the last time your child/children used specialty mental or behavioral health services (such as a psychologist or psychiatrist)?” [response options: within the last 6 months, within the last year, within the last 2 years, within the last 5 years, more than 5 years ago, never]), transformed into a dichotomous variable (yes or no); responding parent health (“In general, how would you rate your health?” [response options: excellent, very good, good, fair, poor]) transformed into a dichotomous variable (better or worse health); and history of firearm violence personally affecting the family as a dichotomous variable (“Has your family been personally affected by gun violence?” [response options: yes, no]).

We adjusted for the aforementioned potential predictors of firearm ownership including self-reported demographics: parent gender dichotomized (male and female) (Parker et al. 2017), parent age transformed into a dichotomous variable (under 35 years and 35 years of age and older) (Parker et al. 2017), income as a percentage of the federal poverty level (FPL; < 100% FPL, 100–399% FPL, 400% + FPL) (Gresham and Demuth 2020), and parent educational attainment (high school or below, some college or technical school, college graduate or above) (Parker et al. 2017). Race and ethnicity, which have demonstrated associations with firearm ownership in prior literature (Parker et al. 2017), were self-reported from the following categories (“non-Hispanic Black,” “non-Hispanic White,” “Hispanic;” and “other,” “two or more,” and “non-Hispanic Asian,” which were combined to “Asian/other race non-Hispanic” for our analysis) (Parker et al. 2017). We also collected parent marital status to explore gender discrepancies in reporting firearms in the home.

### Statistical analysis

We performed all statistical analyses in SAS software version 9.4 (SAS Institute, Inc. Cary, North Carolina). We described the responding population using weighted percentages based on benchmarks from the American Community Survey for race, age, and household income. Using logistic regression, we explored the association of presence of a firearm in the home with predictor variables and demographic and socioeconomic characteristics. Simple models evaluated the unadjusted association of each individual predictor with the presence of a firearm in the home. The final model adjusted for all predictors as well as demographic and socioeconomic characteristics. Marital status was not included in the final model due to collinearity with ACEs (“parent or guardian divorced or separated”) but was used to explore differential reporting of firearms in the home based on gender in a post-hoc chi-square analysis (i.e., whether a gender discrepancy in reporting firearms in the home persisted among married couples). Due to low levels of missingness, we performed a complete case analysis and assumed missing values were randomly excluded. Odds ratios (OR) and adjusted odds ratios (aOR) with 95% confidence intervals (CI) described associations. We analyzed data from June 2021 to November 2022.

## Data Availability

The datasets generated and analyzed during the current study are not publicly available due to participant privacy and raw data cannot be released per the consent form signed by participants. Summary data and descriptive results are available from the corresponding author on reasonable request.

## Abbreviations

ACE: Adverse childhood experience
CI: Confidence interval
OR: Odds ratio
aOR: Adjusted odds ratio
FPL: Federal poverty level
